# Recent Advances in Monitoring Cell Behavior Using Cell-Based Impedance Spectroscopy

**DOI:** 10.3390/mi11060590

**Published:** 2020-06-13

**Authors:** Qusai Hassan, Soha Ahmadi, Kagan Kerman

**Affiliations:** Department of Physical and Environmental Sciences, University of Toronto Scarborough, 1265 Military Trail, Toronto, ON M1C 1A4, Canada; qusai.hassan@mail.utoronto.ca (Q.H.); soha.ahmadi@mail.utoronto.ca (S.A.)

**Keywords:** cell-based impedance, cell culture, electrochemical impedance spectroscopy, high-throughput screening

## Abstract

Cell-based impedance spectroscopy (CBI) is a powerful tool that uses the principles of electrochemical impedance spectroscopy (EIS) by measuring changes in electrical impedance relative to a voltage applied to a cell layer. CBI provides a promising platform for the detection of several properties of cells including the adhesion, motility, proliferation, viability and metabolism of a cell culture. This review gives a brief overview of the theory, instrumentation, and detection principles of CBI. The recent applications of the technique are given in detail for research into cancer, neurodegenerative diseases, toxicology as well as its application to 2D and 3D in vitro cell cultures. CBI has been established as a biophysical marker to provide quantitative cellular information, which can readily be adapted for single-cell analysis to complement the existing biomarkers for clinical research on disease progression.

## 1. Introduction

The process of cell culturing involves the removal of the cells of interest from their host organism, inoculating the cells in an artificial environment, and allowing them to propagate in vitro, until confluency is reached [1]. The process is referred to as “culturing” because after the point of confluency, the cells can be passed and further propagated into future cell lines [1]. Most commonly, cell cultures are used to study cell development, differentiation and propagation as well as genetic manipulation with viral particles and production of biological agents such as vaccines and therapeutic proteins for drug screening and toxicity testing [1,2,3,4]. Cell cultures can either be grown in a suspension, (non-adherent cell lines such as hematopoietic and adipose cells), or on a surface/substrate (adherent cell lines). Generally, adherent cell cultures can be either two-dimensional (2D), in which the cells are inoculated on a petri dish and allowed to grow until confluency in which a monolayer is formed, or three-dimensional (3D), in which the cells are grown on or embedded in a polymeric gel which mimics the extracellular matrix and allowed to grow until confluency [3,4]. Although 3D cell cultures are better models for in vivo cell to cell communication 2D cell cultures are still widely used today due to the relative ease of conducting high-throughput screening (HTS) on them [3,5,6,7], although progress is still being made to apply HTS to 3D cultures [8,9,10].

There are several manual techniques used to assay cell cultures, including hemocytometers to count the number of live versus dead cells [11], phase contrast microscopy to visualize cell structures [12,13], as well as using a light microscope in combination with cell staining [14,15,16] or confocal microscopy to get high resolution images [17,18,19]. Although these assays are well established, they are generally labor-intensive and time consuming [20]. Furthermore, several automated assays have also been developed that use spectrophotometric plate readers in combination with various dyes to measure specific cell properties such as cell proliferation [14,19], DNA quantification [21,22], and myogenic cell differentiation [23]. These staining techniques have the advantage of being rapid and reliable; however, for each property to be studied, a specific stain is required, which ends up consuming several resources [20]. Other automated assays that exist include electronic cell counting using a Coulter counter (which is also based on impedimetric principles), as well as flow cytometry, which can rapidly and reliably count and separate marked cells [19,24,25]. However, these two aforementioned techniques require cells to be in a suspension form [20]. Furthermore, although these assays are well established, they have some limitations. They generally are time consuming, need labeling, and most importantly, these techniques are destructive, and cells need to be sacrificed; therefore, their application is mostly limited to 2D in vitro systems.

Cell-based impedance (CBI) (also known as electrochemical cell–substrate impedance spectroscopy) is an assay that uses the principles of electrochemical impedance spectroscopy (EIS) to measure the dielectric properties of a cell culture. These properties can be used to monitor cell proliferation, metabolism, and viability [26,27]. In principle, any cell type can be studied using CBI, whether they form adherent cell cultures (i.e., cell cultures that are fixed on a substrate), or suspended cell cultures. Furthermore, the technique has proven to be a powerful one to monitor the cell behavior in both 2D and 3D cell culture systems using suspended or adherent cells as the technique is nondestructive and can be used further study cells using other assays such as staining [27,28].

Herein, we review recent advancements in CBI-based technology by focusing on their applications for adherent cell lines. First, the theory of EIS is briefly discussed along with the instrumentation that is commonly used. This is followed by in-depth demonstrations of CBI in the context of areas such as cancer, neurogenerative diseases, toxicology, as well as applications to 3D cell cultures by providing some examples from the recent literature. Finally, we discuss the improvements that have been made in recent years in the field of CBI.

## 2. Instrumentation and Detection Principle of CBI

In terms of commercially available CBI instruments, only two manufacturers currently mass produce the product. Applied Biophysics (Troy, NY, USA) produces the ECIS^TM^ system, which was developed by Giaever and Keese in 1993 [29], and measures frequencies between 25 Hz–100 kHz. ACEA Biosciences (San Diego, CA, USA) manufactures xCelligence^TM^, although it operates using the principles of EIS, uses a specific parameter termed Cell Index, which reported the measured resistance of the adherent cells. Cell index (CI) is defined as:

$$CI=\underset{i=1,\ldots ,N}{\max}\left(\frac{R_{\mathrm{cell}}\left(f_{i}\right)}{R_{0}\left(f_{i}\right)}-1\right)$$

where *R*_cell_(*f_i_*) and *R*_0_(*f_i_*) represent the frequency-dependent resistance of the medium in the presence and absence of cells, respectively, and *N* is number of frequency points for which resistance is measured (usually *N* = 3 for 10, 25, and 50 kHz) [30,31]. Although commercial CBI instruments are available, researchers have designed and fabricated various CBI detection platforms that were tailored for specific applications [28,32,33]. This technology could become one of the essential instruments in cell culture laboratories in the near future.

The instrumentation and the principals of the CBI technology were described in detail by several articles [27,34,35,36,37,38]. Here, we give an overview of the detection principal of the CBI technique. In CBI, the cell culture acts as the primary transducer by which the impedance signal is generated. Cells are cultured on a substrate comprised of an insulating material which is typically glass as it minimizes noise, and a conductive material which is the electrode [39].

**Figure 1:**
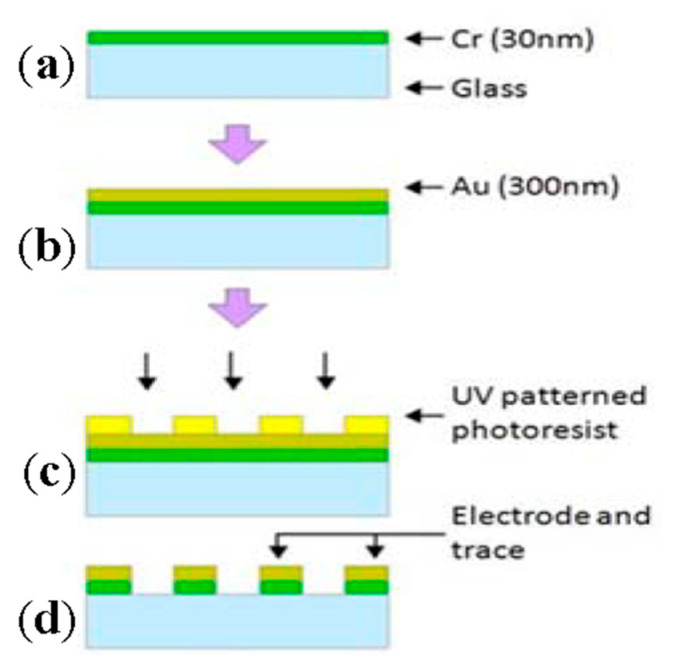
A typical fabrication procedure of the sensing substrate used in cell-based impedance (CBI) set up. (**a**) the glass surface is coated by 30 nm layer of Cr; (**b**) followed by a 300-nm layer of Au; (**c**) and (**d**) Au/Cr layer is subsequently photoetched into interdigitated electrodes. Adapted from Wang et al. [43] under a Creative Commons Attribution License.

A typical fabrication of this substrate is shown in Figure 1. On a glass surface, a 30 nm layer of Cr is sputtered (Figure 1a) followed by a 300-nm-thick layer of Au (Figure 1b), which acts as the electrode. Au is typically used as it is compatible with nanofabrication techniques and can be easily modified [39] (other conductive materials can be used as the electrode material including indium tin oxide [40], Pt [41] and Ni [42]). The Au/Cr layer is subsequently photoetched into interdigitated electrodes (Figure 1c,d). The Au layer can then be further chemically modified depending on the particular sensing needs of the experiment.

**Figure 2:**
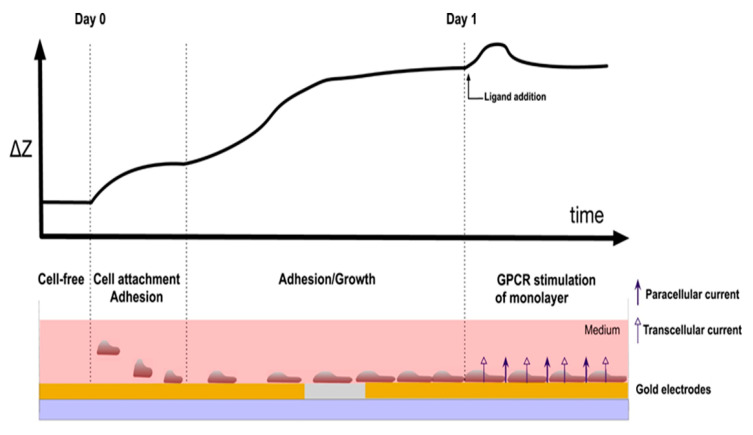
Illustration for the CBI-based assay for the detection of cell adhesion and growth. Once a monolayer of cells was formed, subsequent toxicology and drug screening experiments could be performed. Adapted from Doijen et al. with permission from Elsevier [30].

Figure 2 illustrates the detection principles of CBI [41]. Initially, the cells are deposited in the culture medium in which the electrode substrate sits. The cells then gradually diffuse onto the surface of the electrode and attach to the substrate resulting in a sharp increase in impedance. Initially, the area of contact between the cells and the electrode is small; however, as the cells start to enter the adhesion phase, the cytoskeletons rearrange, and the cell shapes change from spherical to an irregular polygon until the cells are completely attached [36]. Given that the cell membrane is relatively insulating (see next section), as the cells adhere more to the surface, the impedance measured from the electrode gradually increases due to the current passing through the cells being hindered. Once the cells begin to proliferate on the surface of the electrode, the impedance measured will further increase due to the increasing insulating material on the surface of the electrode. At this point, once the cells form a monolayer over the substrate, drug screening or toxicity experiments can be performed while monitoring the changes in impedance. As the growth space is limited and the nutrients in the medium are depleted, the cells will begin to undergo apoptosis and detach from the surface of the electrode, and the measured impedance will then begin to decrease (not shown in figure). As a result, the processes of adhesion, spreading, proliferation and apoptosis can be detected using CBI [36].

CBI provides the advantages of applying rapid, label-free, non-destructive and sensitive measurements of cells with quantitative results in real time [27,29]. Furthermore, cell cultures analyzed with CBI can be subsequently used for other assays such as staining techniques or drug assays [27,29,41]. Another important advantage of CBI is that the experimental results obtained from drug screening and toxicity studies are readily transferrable toward high-throughput screening of drug candidates [41]. However, CBI also presents some limitations, such as the necessity of growing cells on a conductive substrate, which may not be feasible for certain types of cells that would require special conditions for growth. Furthermore, CBI lacks special resolution, which prevents the study of many intracellular processes, although some progress in overcoming this limitation has been made in the past decade [42,44]. Additionally, some intracellular processes generate too low of an impedimetric signal to be detected by conventional CBI instruments, in which case CBI should be coupled with other more sensitive techniques such as fluorescence microscopy [32,41,44]. Lastly, there is a significant demand for the development of standard protocols such that data obtained from independent research groups can be archived and shared. One way to improve this is to collect raw impedimetric data into a library for certain cell types/cell processes [32,41].

## 3. Theory of Electrochemical Impedance Spectroscopy for Cell Analysis

In EIS, a frequency-dependent signal in the form of an AC voltage *U*(*jω*) is applied to the electrode. The resulting current *I*(*jω*) is measured and the impedance is defined as follows [26,36]:

$$Z\left(j\omega \right)=\frac{U\left(j\omega \right)}{I\left(j\omega \right)}=Z_{RE}+jZ_{IM}$$

where *j*^2^ is −1, *ω* is angular frequency, *Z* is the complex impedance, *Z_RE_* is the real component of the impedance (also known as the resistance), and *Z_IM_* is the imaginary component of the impedance (also known as the reactance). The absolute value of the impedance is hence given by [26,36]:

$$\left|Z\right|=\sqrt{{\left(Z_{RE}\right)}^{2}+{\left(Z_{IM}\right)}^{2}}$$

**Figure 3:**
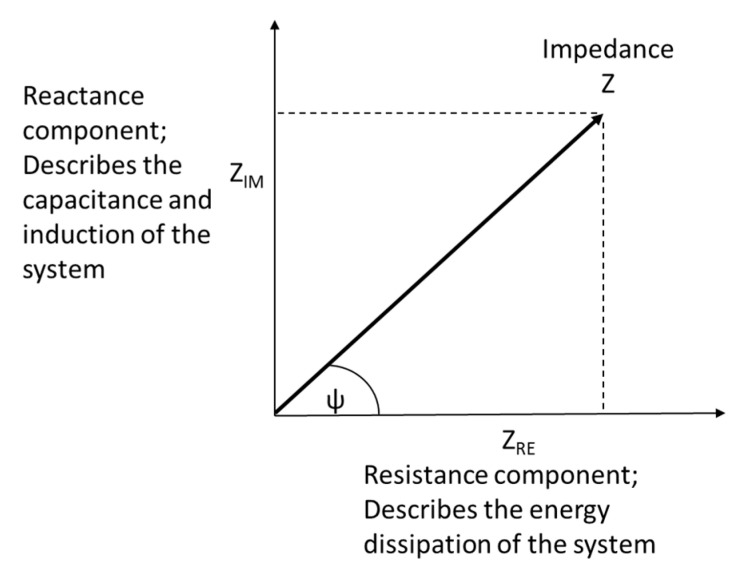
Phasor diagram relating the Cartesian and polar coordinates of impedance in the complex plane.

Given this, the phase shift (*ψ*) between the current and voltage can be calculated as follows (Figure 3) [36]:

$$\psi =arctan\frac{Z_{IM}}{Z_{RE}}$$

As shown in Figure 3, the real component of the impedance describes the energy dissipation of the current as it travels through the material, while the imaginary component of the impedance represents the dielectric capacitance and the induction of the system. The phase shift is therefore dependent on the magnitude of the real and imaginary components of impedance.

**Figure 4:**
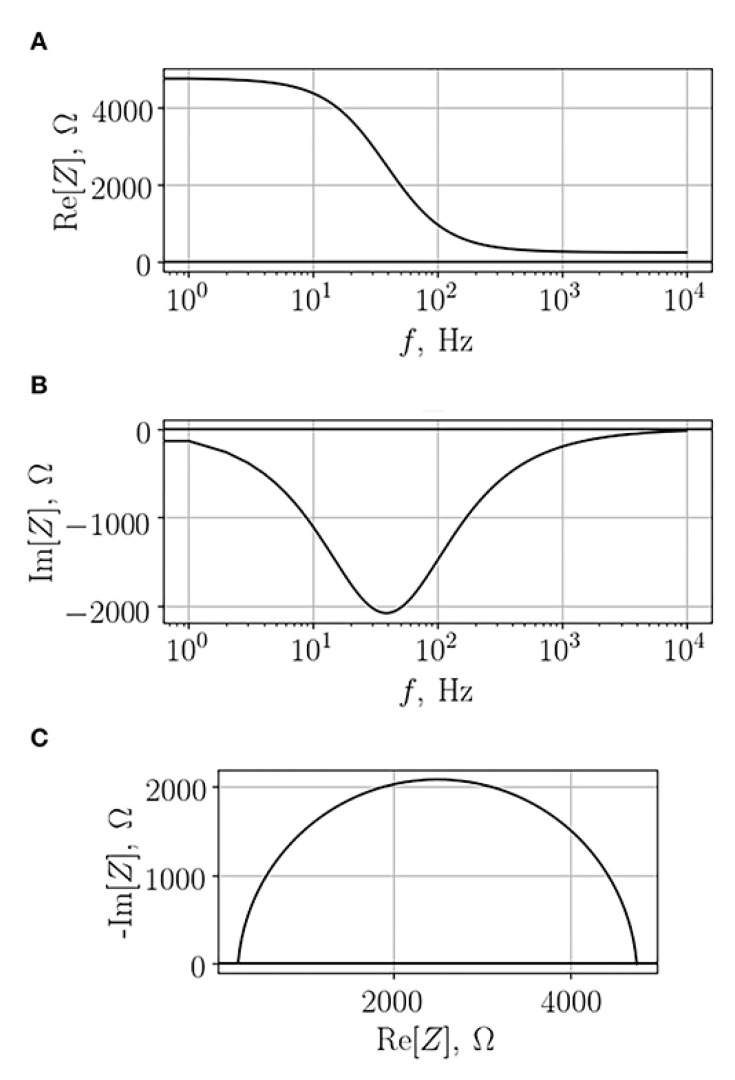
Typical electrochemical impedance spectroscopy (EIS) spectra obtained from a monolayer of cells. (**A**) The real component of the Bode plot, (**B**) the imaginary component of the Bode plot, and (**C**) the Nyquist plot in which the real impedance is plotted against the imaginary one. Adapted from Gerasimenko et al. [36] under Creative Commons Attribution License.

Impedance measurements can be represented as either a Bode plot (Figure 4A,B) in which *Z_RE_* or *Z_IM_* is plotted versus the log of the frequency range measured, or a Nyquist plot (Figure 4C) in which *Z_RE_* is plotted against *Z_IM_*. As mentioned previously, the real and imaginary impedance vary depending on the frequency applied, hence a range of frequencies is typically swept.

**Figure 5:**
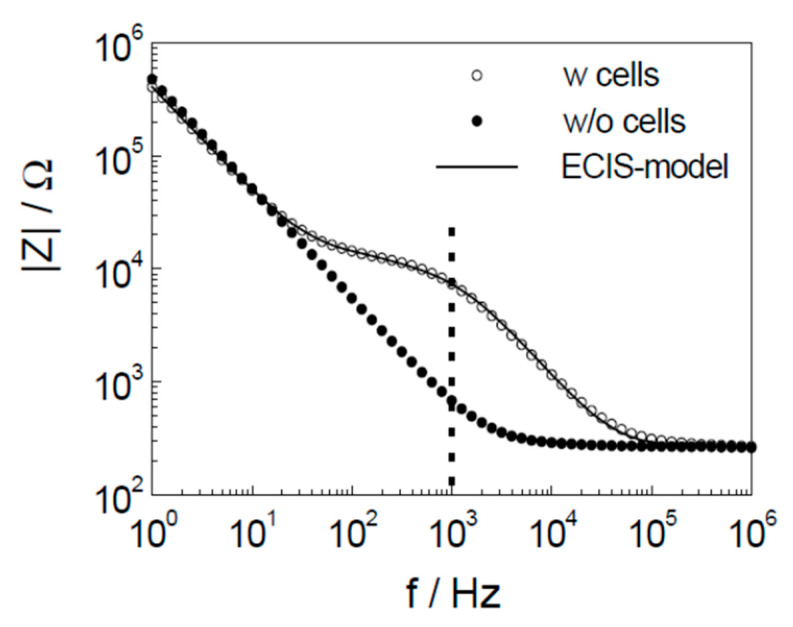
CBI spectra with and without cells performed on a gold electrode verifying that frequencies outside of 10 Hz–100 kHz were independent of cell properties. Reprinted from Arndt et al. [45] with permission from Elsevier.

Given that the impedance of a system depends on the applied frequency, generally a range of frequencies are scanned. In the case of CBI, the frequency ranges swept tend to be between 10 Hz–100 kHz as Arndt et al. [45] showed by monitoring the apoptosis induced changes in cell shape that the impedance measured at frequencies below this range was due to the electrode/electrolyte interaction and the impedance measured outside of this range was due to the sum of the contributions from the culture medium, the constriction resistance of the working electrode, and the wiring of the system (Figure 5) [34,45]. Hence, the impedance measured outside of the 10 Hz–100 kHz range was independent of the cell properties.

**Figure 6:**
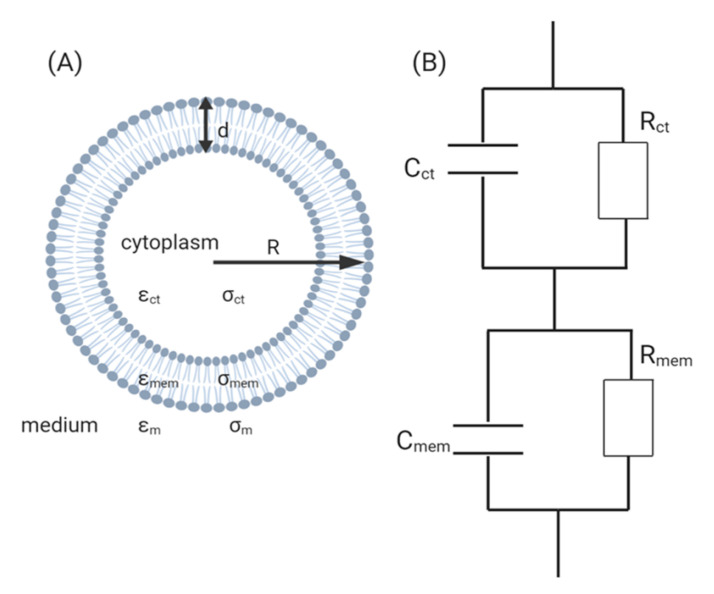
(**A**) A diagram of the electric model of the cell according to Maxwell’s theory of mixtures. (**B**) Equivalent circuit of the single cell model [26,48].

The most commonly accepted equivalent circuit model of biological cells is the “single shell”, which simplifies the components of a cell and assumes that the cytoplasm is a conductive sphere encapsulated by a non-conductive sphere which is the lipid-bilayer (Figure 6A) [26,34,46,47]. It is worth noting that this model only applies to animal cells, since bacteria and plant cells possess an additional cell wall and hence, the model needs to be modified to take that into consideration [35].

Using this model, the complex permittivity of the cell can be derived using Maxwell’s mixture theory [34]:

$$\varepsilon_{\mathrm{cell}}=\varepsilon_{\mathrm{mem}}\frac{\rho^{3}+2\left(\frac{\varepsilon_{\mathrm{ct}}-\varepsilon_{m}}{\varepsilon_{\mathrm{ct}}+2\varepsilon_{m}}\right)}{\rho^{3}-\left(\frac{\varepsilon_{\mathrm{ct}}-\varepsilon_{m}}{\varepsilon_{\mathrm{ct}}+2\varepsilon_{m}}\right)}$$

where $\rho =\frac{R}{R-d}$, *ε*_cell_, *ε*_mem_, *ε*_ct_, and *ε*_m_ represent the complex permittivity of the cell, membrane, cytoplasm and medium, respectively. *R* is the radius of the cell and *d* is the length of the lipid, where *σ*_mem_, *σ*_ct_, and *σ*_m_ represent the conductance of the membrane, cytoplasm and medium, respectively.

Both the conductivity and permeability of the cell can be assumed to be of the same order of magnitude as the surrounding growth medium. However, given that the lipid bilayer acts as an insulator, the permeability and conductance of the membrane will be significantly smaller than that of the cytoplasm such that *ε*_m_ << *ε*_ct_ and *σ*_m_ << *σ*_ct_. Indeed, it has been reported that the conductance of the membrane and cytoplasm typically measure at 0.3 mS/cm^2^ and 0.005 S/cm^2^, respectively, while the specific capacitance of the membrane measures at 1 µF/cm^2^ [34,48]. Only in the case of cell death when the cell membrane begins to form porous structures would *ε*_m_ and *σ*_m_ approach the values of *ε*_ct_ and *σ*_ct_, respectively [26].

Since the study of conductance and permeability require complex mathematical equations as shown above, electrochemists prefer to lump these parameters together. For example, conductance can be considered as resistance (*R*), as follows:

$$\left|Z_{RE}\right|=R\;\left|Z_{IM}\right|=0$$

Additionally, permeability can be considered as capacitance using the following equations:

$$\left|Z_{RE}\right|=0\;\left|Z_{IM}\right|=\frac{1}{j2\pi fC}$$

where *f* is the frequency applied and *C* is the capacitance.

## 4. Applications

CBI techniques can be used in a wide range of research fields for various applications. In this section, we provide an outlook into the recent advances of CBI applications in research efforts towards cancer, neurodegenerative diseases, and toxicity as well as discussing the ability of the CBI system to evaluate the 2D and 3D in vitro models. Other important applications of the CBI techniques have been discussed by Benson et al. [49]. The CBI system can also be used as a tomography sensor for cell imaging [50,51,52,53]. Combining CBI with microfluidic systems and flow cytometry provided the development of miniaturized devices for automated bioanalysis [54,55,56,57,58,59,60]. Although these devices may be far from high throughput applications, they have the advantages of simplicity, low sample size that make them valuable tools for fundamental and applied research.

### 4.1. Cancer Research

Cancer is one of the leading causes of death worldwide, with a rapid incidence and mortality growth [61,62]. Given that more than a million new cancer cases are diagnosed each year, there is an emerging need for developing early diagnostic tools and effective therapeutic strategies. In vitro studies are in the forefront of drug design and they are considered as an essential part of cancer research [63,64]. Studying cell behavior not only provides valuable information about drug potency and efficacy but is also critically important to investigate drug resistance [64,65]. In vitro studies are also performed to investigate cell migration and invasion, which are significantly implicated in cancer metastasis [66,67,68]. Therefore, it is crucial to use accurate and reliable techniques to study cell behavior in cancer research. The combination of advanced cell analysis techniques with in vitro cancer models that mimic the in vivo pathological conditions is a key for successful therapeutic strategies. There is growing interest in using CBI instruments due to their advantages and ability to perform real-time label-free analysis [27,34,38,69,70]. In the last few years, several novel strategies have been introduced to study cancer cell behavior using the CBI [52,71,72,73,74,75].

**Figure 7:**
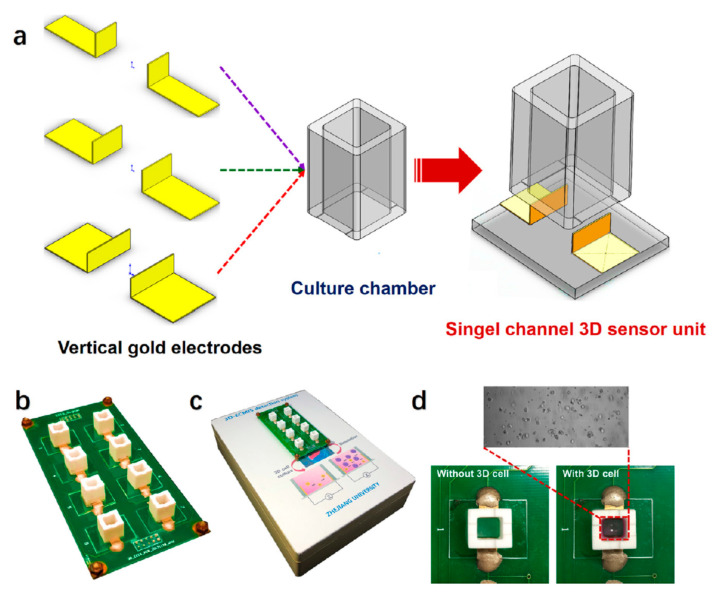
Schematic of the different parts of 3D electric cell/matrigel-substrate impedance sensing (3D-ECMIS): (**a**) vertical gold electrodes in a single channel, (**b**) arrangement of 8 channels on the scaffold, (**c**) picture of the eight channels in the detection system, and (**d**) images of the 3D cell-based biosensor before and after seeding the living cells. Reprinted from Pan et al. [76] with permission from Elsevier.

Wang and coworkers [76] introduced an impedance sensor for real-time cell viability analysis in a 3D in vitro model called 3D electric cell/matrigel-substrate impedance sensing (3D-ECMIS). The 3D-ECMIS was fabricated with eight individual sensor channels. Each channel contained a pair of gold electrodes, a culture chamber, and a glass substrate (Figure 7). To perform the analysis, an initial potential of 30 mV was applied to the electrodes and the impedance was measured in the range of 0.1–50 kHz every 10 min for 96 h. They examined the anticancer activity of three different drugs (ovarian/breast anti-cancer drug, Taxol; broad-spectrum anti-cancer drug, cisplatin; and liver anti-cancer drug, sorafenib) using the 3D in vitro model of human hepatoma cells (HepG2) which were encapsulated in a matrigel scaffold. They showed that the developed impedance sensor can be used to improve the accuracy of drug screening in cancer research [76].

**Figure 8:**
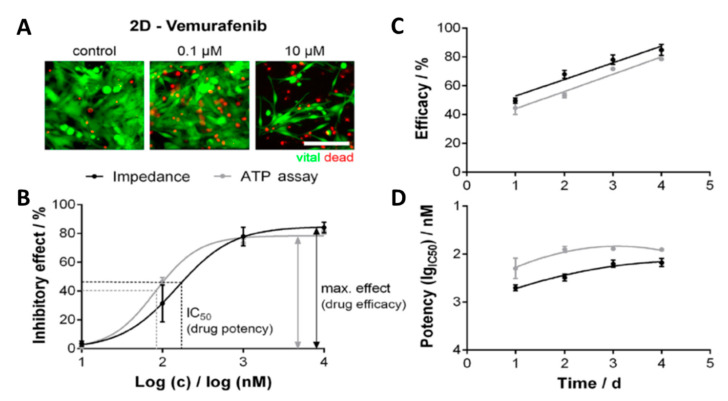
Comparison of the results obtained from the impedance sensor and the Adenosine triphosphate (ATP) assay. The serine/threonine-protein kinase B-Raf (BRAF) inhibitor vemurafenib was applied to 2D mutated melanoma in vitro models. (**A**) Fluorescence images of the melanoma cells with and without treatment with vemurafenib. (**B**) Normalized concentration-response curves, (**C**) efficacy plotted over time, and (**D**) potency plotted over time obtained from the impedance sensor (black line) and ATP assay (gray line). Reprinted from Seidel et al. [73] with permission from Elsevier.

Wang and coworkers [71] also developed a multi-dimensional microgroove impedance sensor (MGIS) for real-time analysis. A silicon wafer was used to fabricate the MGIS with a gold electrode. The MGIS was utilized for high-throughput pharmacokinetic analysis of drug candidates for lung cancer. They used a 3D in vitro model of lung cancer to improve the predictability and reduce the false positive results of drug testing. The developed MGIS was tested using adenocarcinomic human alveolar basal epithelial cells (A549), human hepatoma cells (HepG2), and Madin–Darby canine kidney cells (MDCK) that were encapsulated in matrigel to provide a 3D in vitro system. They used cisplatin to evaluate the drug efficacy as well as testing drug synergy using equimolar of cisplatin with gemcitabine and cisplatin with pemetrexed. They validated the MGIS performance by testing its stability, electrical properties, reproducibility, and long-term reliability [71]. Seidel et al. [73] also proposed a multidimensional impedance platform to test anticancer activity in a single and combination treatment for melanoma cancer. They used tissue derived from a patient to develop 2D and 3D cell culture model for melanoma cancer. The efficacy and pharmacokinetics of different anticancer drugs against the melanoma cells were tested using the developed sensor, which was a multielectrode and microcavity-based multidimensional impedance platform. Impedance spectra were recorded before and after drug treatment between 5 kHz and 5 MHz. The results were compared with adenosine triphosphate (ATP) cell viability bioassay, to evaluate the performance of the developed sensor (Figure 8) [73].

**Figure 9:**
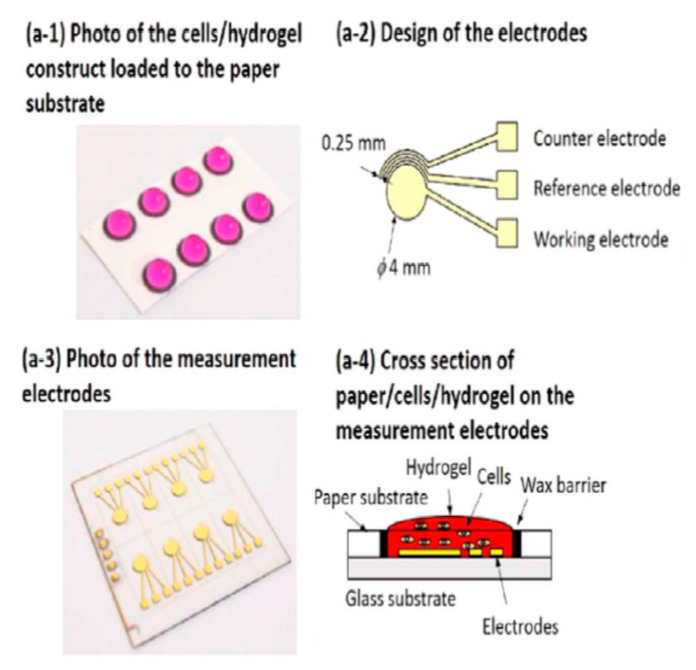
Schematic procedure of fabrication of the paper-based CBI sensor. Reprinted from Li et al. [77] with permission from Elsevier.

The cost of CBI devices can be reduced using a paper substrate to fabricate the impedance sensor. Lei et al. [77] reported a simple and cost-effective paper-based impedance sensor (Figure 9) that was developed to test the chemosensitivity of hepatoma cells in a 3D in vitro system [77]. The developed sensor was fabricated with paper and glass substrates. A 2 × 4 array of 6 mm diameter circles was drawn on a regular filter paper using a solid wax printer. The wax on the paper was fully melted to provide a hydrophobic barrier, which provided microwells for the cell culture. The glass substrate was used for the three-electrode impedimetric setup, and standard microfabrication was used to fabricate a Cr/Au working electrode. Then, the paper substrate was mounted on the glass substrate for cell viability analysis by measuring impedance between 0.1 to 100 kHz with an electric potential of 0.1 V_rms_, which was reported harmless to the cells. The 3D in vitro model was developed by encapsulating the cells in 0.5% agarose hydrogel. The biosensor was used to evaluate the effect of two anticancer drugs (doxorubicin and etoposide) against Huh7 and Hep-G2 hepatoma cell lines. The results showed that doxorubicin was a stronger anticancer agent compared to etoposide, and Huh7 cells had a higher drug resistance than the Hep-G2 hepatoma cells [77].

Adcock et al. [78] applied a real-time cell impedance sensing (RT-CES) device to show the potential of CBI systems in personalized medicine. To prove the concept, six different chemotherapeutic drugs were tested against three prostate cancer cell lines and a normal prostate cell line. Cell viability was monitored during the time after treatment with the different drugs to identify the most potent drug for each type of cancer cells, which did not indicate any drug resistance for the tested anticancer drugs [78].

**Figure 10:**
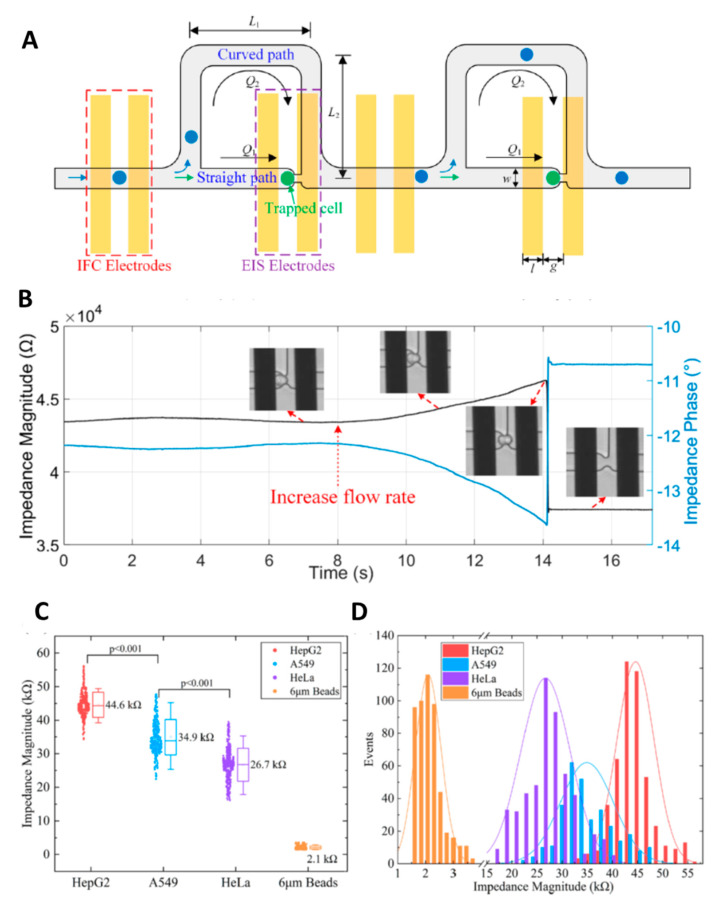
(**A**) Schematic diagram of the microchip structure depicting the cells passing/trapping for impedance flow cytometry (IFC)/ electrochemical impedance spectroscopy (EIS)sensing. Cells would pass through the IFC channel and then be trapped in the EIS site. (**B**) The dynamic response of EIS-based impedance magnitude and phase when the flow rate was kept as 10 nL/min for a while and then increased to 10 μL/min (*f* = 1 MHz). (**C**) The average impedance magnitudes of HepG2, A549, HeLa cells and 6 μm beads, showing significant difference (*p* < 0.001). (**D**) The impedance magnitudes of cells and beads exhibit Gaussian distribution in population. Reprinted from Feng et al. [53] with permission from American Chemical Society.

Several studies showed the capability of CBI systems to differentiate cancer cells using different designs [53,79,80]. A microfluidic impedance system was designed to detect leukemia cells, which showed the ability to differentiate red blood cells from the leukemia cells. The geometry of the microchannels, as well as conductivity of the carrier solution, excitation voltage, and particle size, were optimized to improve the precision and sensitivity of the microfluidic system [79]. The authors claimed that this cost-effective and reusable device could be used as a high throughput technique to diagnose leukemia and other hematological diseases like malaria and anemia [79]. Fing et al. [53] also developed a microfluidic system, which was a combination of impedance flow cytometry (IFC) and EIS to differentiate three different cancer cell lines including HeLa, A549, and HepG2. They tested the performance of IFC and EIS individually as well as in the combination setup, which showed the system with the combination of IFC and EIS had a higher efficiency than each individual technique. In the combined device, cells were passed by the IFC and trapped by the EIS part of the system. The authors showed the ability of IFC to classify HeLa, A549, and HepG2 when compared to microbeads as the reference group (Figure 10). Their results showed that at 1 MHz frequency the overall impedance was dominated by size and electrical property of the single cells, which allowed the system to differentiate the cell types [53]. Another study demonstrated the ability of a novel microfluidic impedance device to differentiate cancer cells, bead-cell aggregates, and bare magnetic beads [80]. The multi-frequency microfluidic impedance cytometry device was fabricated with two gold electrodes on a glass wafer substrate and polydimethyl siloxane micro-channels, which were combined with an immuno-magnetic separation technique. The device was capable of quantitatively detecting 1.5 million cells/mL, which could be used to detect dissociated cancer cells obtained from a tissue biopsy. However, the detection limit of the developed device was not enough for detecting circulating tumor cells in blood [80].

**Figure 11:**
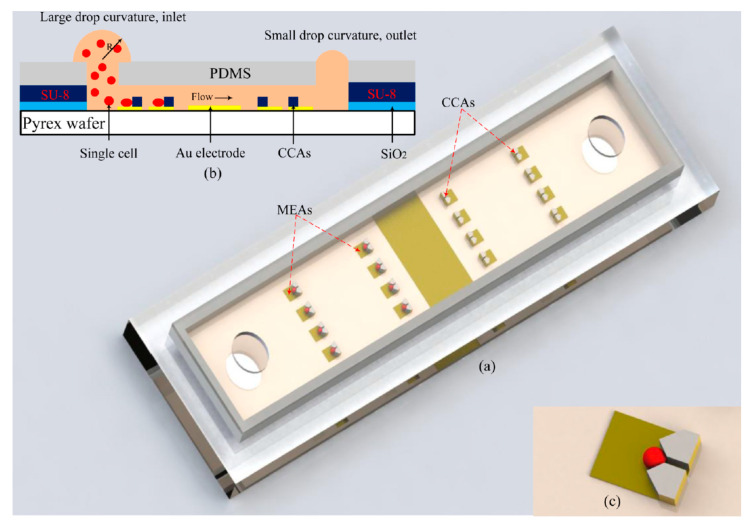
(**a**) 3D image of the sensor chip, which consists of three main parts: microelectrode arrays (MEAs), cell capture arrays (CCAs), and a microfluidic channel with an inlet and an outlet. (**b**) Cross-sectional view of the MFC with a mechanism of passive pumping. (**c**) A V-shaped structure for single cell trapping. Reprinted from Nguyen et al. [54] with permission from American Chemical Society.

Cancer metastasis is an important field in cancer research, in which detached circulating tumor cells (CTCs) spread to new areas of the body through the lymph system or blood stream resulting in metastatic tumors [81,82]. A recent publication reported the development of a disposable microfluidic impedance device with two constriction channels and embedded electrodes that detected and enumerated cancer cells in the blood [83]. CTCs found in the blood play an important role in cancer metastasis and the formation of the secondary tumors. CTCs also can be used for diagnosis and monitoring the cancer progress. Ghassemi et al. [84] used the developed microfluidic impedance device to provide information about the biophysical characteristics of breast and prostate cancer cells. They detected and differentiated breast and prostate cancer cells that were spiked into murine blood [83]. Cell migration, which is an important factor that could initiate the cancer metastasis, could be monitored by CBI techniques as well. A 96-well high-density microelectrode array for impedimetric analysis of breast cancer cell line and two malignant melanoma cell lines were employed to study cell migration [72]. The results obtained from the impedimetric data were also supported by microscopy imaging and transwell assays [72]. Nguyen et al. [54] designed an electrical cell-substrate impedance sensing (ECIS) chip using the Boyden chamber design to study the kinetics of migration and invasion of single cancer cells. The designed chip consisted of three main parts: microelectrode arrays (MEAs), cell capture arrays (CCAs), and a microfluidic channel with an inlet and an outlet (Figure 11). The injected cells were hydrodynamically trapped by the CCAs in a 2D or 3D scaffold for the real-time study of cell behavior by MEAs. The impedance measurements were performed with an alternating voltage of 10 mV between a frequency range of 100 to 10^6^ Hz. The real-time analysis was carried out at 4 kHz with an alternating voltage of 10 mV. The authors claimed that the rapid variation of impedance magnitude can be correlated with cell migration. The ability of the designed chip to monitor cell migration was tested using a 3D model of two different breast cancer cell lines (MDA-MB-231 and MCF-7). It was shown that while impedance remained stable for MCF-7, the impedance decreased during the time when MDA-MB-231 was mounted on the chip, which indicated that MCF-7 was less metastatic than MDA-MB-231 cells [54].

### 4.2. Neurodegenerative Diseases

Compared to the cancer research, there is less reported research that utilizes CBI devices to study neuron behavior and neurodegenerative diseases. These few studies demonstrated the capability of the CBI devices to monitor the behavior of neurons in physiological and pathogenically conditions using 2D and 3D in vitro models [84,85,86,87,88,89,90,91].

One of the early studies that showed the capability of using CBI devices in neurodegenerative research was performed by Robitzki and coworkers [85]. They used a multielectrode array (MEA) with 60 square-like indium tin oxide (ITO) electrodes to study the differentiation of human neuroblastoma SH-SY5Y cells. They investigated the effect of staurosporine, a kinase inhibitor, on differentiated neuronal phenotype. The results obtained from the device was correlated with common bioassays such as western blot and immunocytochemistry analysis [85].

Tau hyperphosphorylation has been the subject of numerous researchers due to its implication to a group of neurodegenerative diseases called tauopathies [92,93]. A real-time EIS system with a multielectrode array was applied to monitor retrograde neurite degeneration in tauopathies by inducing tau hyperphosphorylation [84]. The ESI system was fabricated with 60 nanocolumnar structured titanium nitride electrodes in an 8 × 8 grid platform, which was coated with nitrocellulose. To mimic the pathogenic condition, tau hyperphosphorylation was induced in hippocampal slices, originated from Sprague Dawley rat pups, using okadaic acid. The experiments were performed after five days culturing at 37 °C in a humidified atmosphere in a closed chamber. The impedance spectra were recorded by applying an alternating voltage of 10 mV in a frequency range from 500 Hz to 5 MHz every 15 min using at least 30 electrodes. The results obtained from this study indicated that neurite degeneration consisted of two separate phases, in which the relative impedance measured was above the control level in the first phase while in the second phase, the relative impedance measured declined below the control levels. Furthermore, ESI experiments indicated that both degeneration phases could be prevented by suppressing tau hyperphosphorylation using a kinase inhibitor. This study illustrated the potential application of CBI devices to investigate the molecular mechanism of tau-based therapeutic approaches in Alzheimer’s disease [84].

The CBI system was also used to study the neural signals using an in vitro model [87,88]. Zuo et al. [87] designed a multiple microelectrode array, with 105 electrodes, to stimulate and record the extracellular neural signals of mouse brain slices. The electrodes were manually penetrated into the brain slices to record the neural field potential of the tissue [87]. Recently, a diamond 3D microelectrode array, with a hexagonal shape and high electrode density was developed for neuronal stimulation. An in vitro model of rat retina was used to test the performance of the device, which showed that the 3D diamond electrodes could provide a localized stimulation of retinal ganglion cells [88].

**Figure 12:**
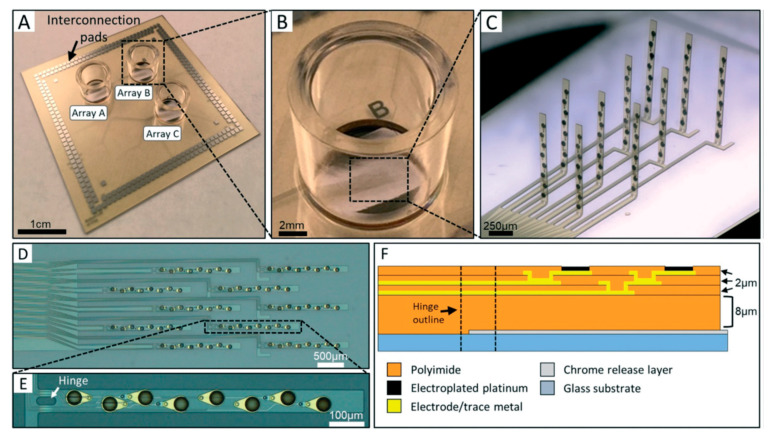
(**A**) Picture of 3D flexible microelectrode array (3DMEA) completed device. (**B**) Close-up image of a single cell culture well. (**C**) Light micrograph of a single 3DMEA post-actuation. (**D**) Brightfield image of one MEA array containing 10 probes and 80 total electrodes prior to actuation. (**E**) Detail of one probe containing 8 electrodes. (**F**) Cross-sectional cartoon showing material stack of microfabricated probe and relative location of hinge (not to scale). Reprinted under Creative Commons Attribution 3.0 Unported Licence [86].

A recent work by Soscia et al. [86] reported the first attempt to monitor a 3D co-culture in vitro model of the brain using a 3D flexible microelectrode array (3DMEA). Figure 12 illustrates different parts of the designed 3DMEA, which consisted of 10 probes, 80 electrodes, and 256 channels. Each probe fabricated with 8 electrodes on a glass substrate (Figure 12F). The fabricated device was integrated into a standard commercially available electrophysiology hardware. To test the 3DMEA, human induced pluripotent stem cell (hiPSC)-derived neurons and astrocytes were co-cultured and encapsulated in collagen-based hydrogel inside the 3DMEA chamber. The cell behavior in the 3D co-cultured system was monitored for 45 days to study the electrophysiological characterization of 3D networks of hiPSCs and astrocytes [86]. The presented 3DMEA had the potential to be tailored for a 3D in vitro model of different neurodegenerative diseases with a vast range of applications. In addition, as the authors suggested, resolution of the system could be improved by increasing the electrode density, which might also provide better coverage of the 3D neuronal network. We envisage that this study may open a new direction to study the neurodegenerative diseases using 3D in vitro co-culture models.

### 4.3. Toxicity Research

**Figure 13:**
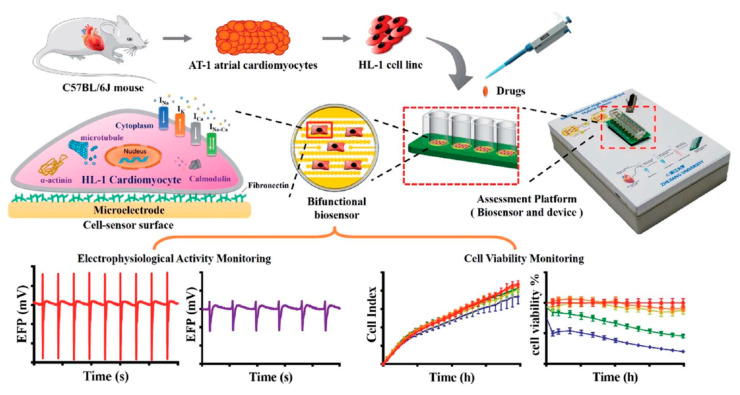
Schematic diagram of the drug-induced cardiac side-effect assessment platform with the CBI system and long-term electrophysiology recording platform. Reprinted from Gu et al. [96] with permission from Royal Society of Chemistry.

Toxic side effects are an important part of the drug design that need to be investigated for any drug candidates [94,95]. CBI devices are powerful tools to study the toxicity and the side effects of the drug candidates using in vitro models [96,97]. A study by Gu et al. [96] investigated the short and long term drug-induced cardiac side-effects of nifedipine and vinblastine using a cardiac muscle cell line (HL-1). Vinblastine is a chemotherapeutic medication for many types of cancer, which inhibits cell proliferation. While nifedipine, which is an inhibitor of L-type voltage-gated calcium channels, widely prescribed to manage high blood pressure. Figure 13 illustrates the workflow of this study, which started by obtaining a primary culture of HL-1 cardiac muscle cells from a mouse model and continued by monitoring the cell viability using impedance and long-term electrophysiology recording platform. The drug-induced cardiac side-effects were evaluated based on the cell viability, cell index (CI), and electrophysiological activities of the HL-1 cells, which indicated that vinblastine induced short-term side effects by reducing the cell viability to 20%, while cell viability after treatment with nifedipine was more than 80% compared to the control samples [96].

Kustermann et al. [98] proposed an impedance-based approach to differentiate cytostatic from cytotoxic drugs by studying the kinetic effects of the drugs on a NIH 3T3 fibroblast cell line (NIH 3T3) using a commercial CBI device over 5 days. The CI obtained from the impedance correlated with cytotoxicity and cell proliferation. The authors proposed an algorithm to differentiate cytostatic, cytotoxic and non-toxic compounds based on the shape of the impedance spectrum. They applied their approach to evaluate 37 compounds [98].

**Figure 14:**
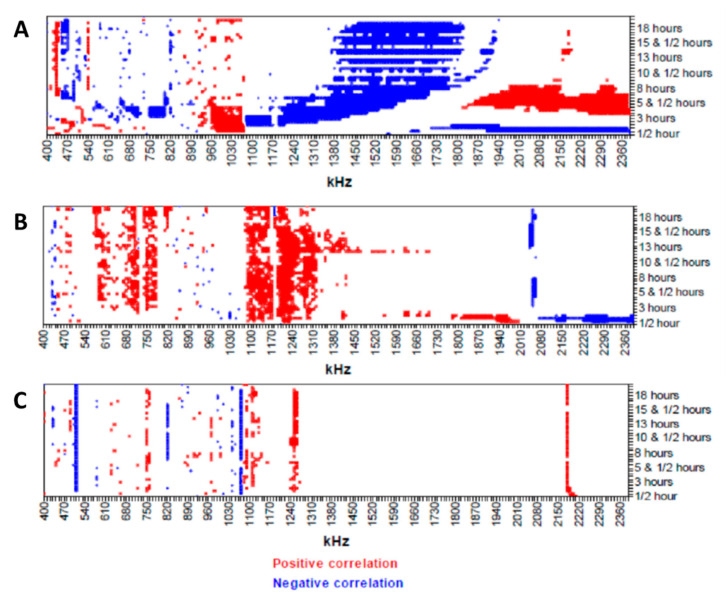
Toxin map of endothelial cells (ECV304) response to (**A**) dimethyl sulphoxide, (**B**) saponin, and (**C**) cadmium chloride treatment during 24 h at the frequency range 400–2400 kHz. Reprinted from Gasser et al. [99] with permission from Elsevier.

Gassar et al. [99] introduced a novel approach, which combined EIS and data analysis to provide a toxin map (Figure 14). They designed a non-contact multi-electrode array, which could be assembled in a standard culture plate for real-time analysis of cytotoxicity. The performance of the device was tested by monitoring the cytotoxicity of three different compounds (dimethyl sulphoxide, saponin, and cadmium chloride) against endothelial cells (ECV304). Figure 14 shows the toxin maps of these compounds, which were obtained from EIS measurements over 18 h. The significant differences in the toxin maps emphasized the differences between the toxicity effects of these three compounds on the endothelial cells. The authors suggested that these toxin maps could be correlated with morphological and physiological changes in the cells due to the different mechanism of the toxicity of each compound [99].

### 4.4. 2D and 3D In Vitro Studies

Although 2D cell culture is a well-established approach, which has been widely used for more than hundred years in many areas of research, it does not accurately represent the biological environment of cells in the tissue [4,100]. 3D cell cultures overcome the limitation of the 2D in vitro model by providing a more reliable environment that mimics the cell behavior in tissues. In the 3D in vitro model, cells grow in all directions into an extracellular matrix or artificial hydrogel, which allows for cell-to-cell communication that is more comparable to the in vivo environment [4,100]. Compared to 2D cell cultures, 3D in vitro models provided better cell differentiation, realistic proliferation rates, gene/protein expression, and an accurate response to drug and mechanical stimulation, which might improve the rate of success in animal studies for drug screening [4,100]. Even though the number of publications on the 3D in vitro models dramatically increased in the last decade, challenges in applying the common bioassays limits its application. There is a limitation to taking images with an inverted microscope due to the 3D nature of the cells. Although a confocal microscope provides 3D images, it remains challenging when cells are embedded in a thick gel which would destructively interfere with light. Application of spectroscopic techniques are limited since detaching the cells from the gel and labelling them with a fluorophore remain challenging. Also, the gel may interfere with the spectroscopic measurements. The non-destructive nature of the CBI makes this technique an ideal strategy to study the 3D cell models. Moreover, the real-time analysis of CBI sensors will be a great asset since the 3D models are expensive to develop, and for most of the common bioassays, cells need to be sacrificed [35,37,101]. Gerasimenk et al. [35] provided an overview of the CBI setup for the 3D in vitro systems and discussed the advantages of this technique for real time analysis. Challenges to apply CBI technique for analyzing 3D in vitro systems were discussed by De León and colleagues [37]. They emphasized the importance of the electrodes in the CBI setup, in which the electrodes should not disturb the 3D cell culture system in order to provide a real-time quantitative analysis.

**Figure 15:**
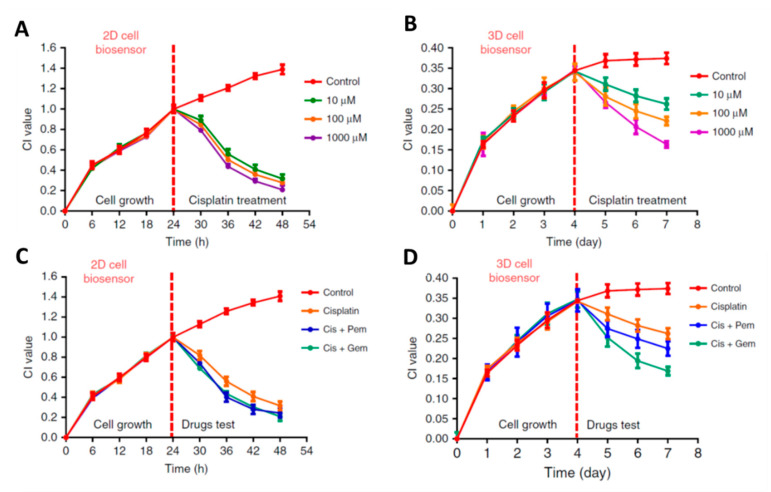
Real-time monitoring of the effects of cisplatin (Cis) on lung cancer cells in (**A**) 2D in vitro and (**B**) 3D in vitro models. Evaluation of the combination treatment of Cis with gemcitabine (Gem) and Cis with pemetrexed (Pem) on lung cancer cells in (**C**) 2D in vitro and (**D**) 3D in vitro model. The cell index (CI) values were calculated from the developed CBI sensor. With permission under the terms of the Creative Commons CC BY license from Springer Nature [71].

Pan et al. [71] compared the 2D and 3D in vitro models for drug screening using a designed CBI system. They evaluated the cytotoxic effects of cisplatin as well as in combination with gemcitabine/pemetrexed against a lung cancer cell line (A549). Figure 15 illustrates the real-time monitoring of these evaluations for 2D (Figure 15A,C) and 3D (Figure 15B,D) in vitro systems. The significant differences in the kinetics and profile effects of 2D and 3D models emphasized the differences between the two models. While the 2D model showed the cytotoxic effects of cisplatin alone and in combination with two other drugs after 24 h, no effects were observed until 4 days after the treatment in 3D in vitro model. Notably, the 3D in vitro model was able to differentiate the different concentrations of cisplatin (Figure 15B) as well as differentiating between combination treatments (Figure 15D) [71].

**Figure 16:**
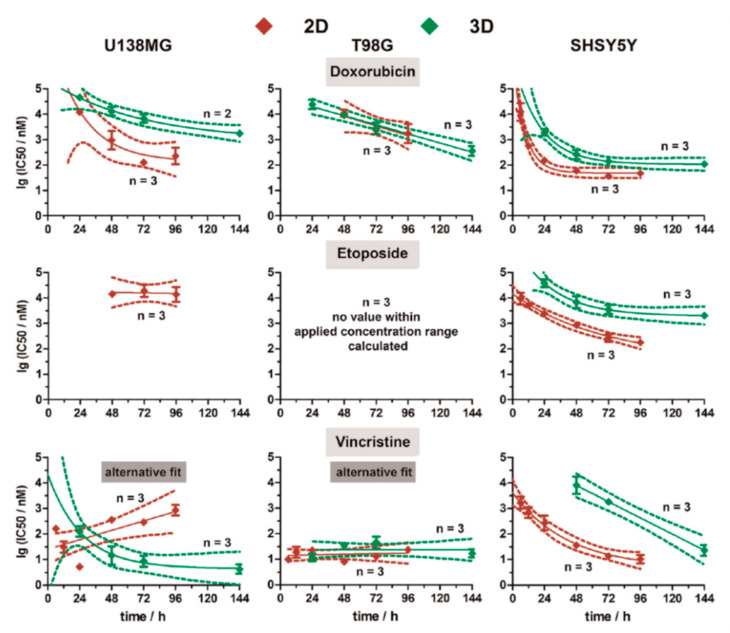
Comparison of IC_50_ values (vincristine, doxorubicin, and etoposide) obtained from real-time impedimetric measurements on neuroblastoma (SH-SY5Y) and glioblastoma (T98G, and U138MG) 2D and 3D cell culture models. Reprinted from Eichler et al. [102] with permission from Elsevier.

Robitzki and coworkers [73,102] also reported on the differences between 2D and 3D in vitro models in two different studies, in which both studies showed a meaningful difference in the pharmacokinetics of the tested drug. In one of their studies, the cytotoxicity of the BRAF inhibitor vemurafenib was tested against melanoma 2D and 3D models using a multidimensional impedance platform [73]. Their results indicated meaningful differences in efficacy and potency of the vemurafenib obtained from these two in vitro models. They also evaluated the cytotoxicity of three drugs (vincristine, doxorubicin, and etoposide) against SH-SY5Y (neuroblastoma) cells, T98G, and U138MG (human glioblastoma) cells using a 96-well multielectrode array-based impedimetric platform [102]. The experiments were performed on both the 2D and 3D in vitro models of the cells. Figure 15 shows the real-time monitoring of the cytotoxicity of the three tested drugs. While there were meaningful differences in the cytotoxicity profile of cells between 2D and 3D SH-SY5Y in vitro models, the differences for the T98G cells were negligible (Figure 16) [102].

## 5. Conclusions

In conclusion, this review article detailed the theory and recent applications of CBI for research in the areas of cancer, neurodegenerative diseases, toxicology, as well as its applications to both 2D and 3D in vitro cultures. CBI is rapidly becoming an established approach to non-destructively evaluate and perform the quality control of cell cultures with quantitative and sensitive data that can be easily adapted for single-cell analysis. CBI also provides complementary information for label-free phenotypic assays when combined with in vitro disease models. However, some limitations remain to be solved such as the challenges of growing the cells on a conductive substrate and the lack of spatial resolution. This technique is used in a widespread of applications to analyze the effects of various therapeutic agents, nanoparticles and toxins for the pharmaceutical and environmental studies.
